# Supplementary material for: Molecular Characterization of BRCA1 c.5339T>C Missense Mutation in DNA Damage Response of Triple-Negative Breast Cancer
Source: Cancers (Basel). 2022 May 13;14(10):2405. doi: 10.3390/cancers14102405 (PMC9139203; doi:10.3390/cancers14102405)
Supplement: Supplementary file 1 [file cancers-14-02405-s001.zip › cancers-1692757-supplementary.pdf]

cancers MDPI

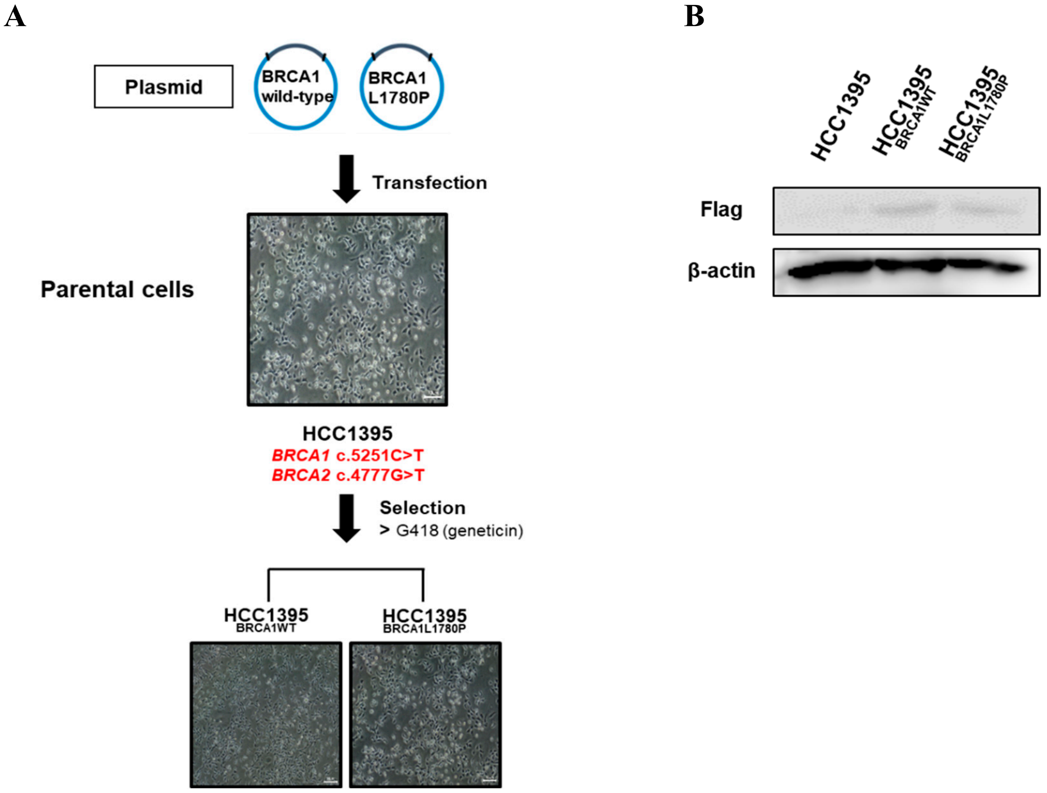

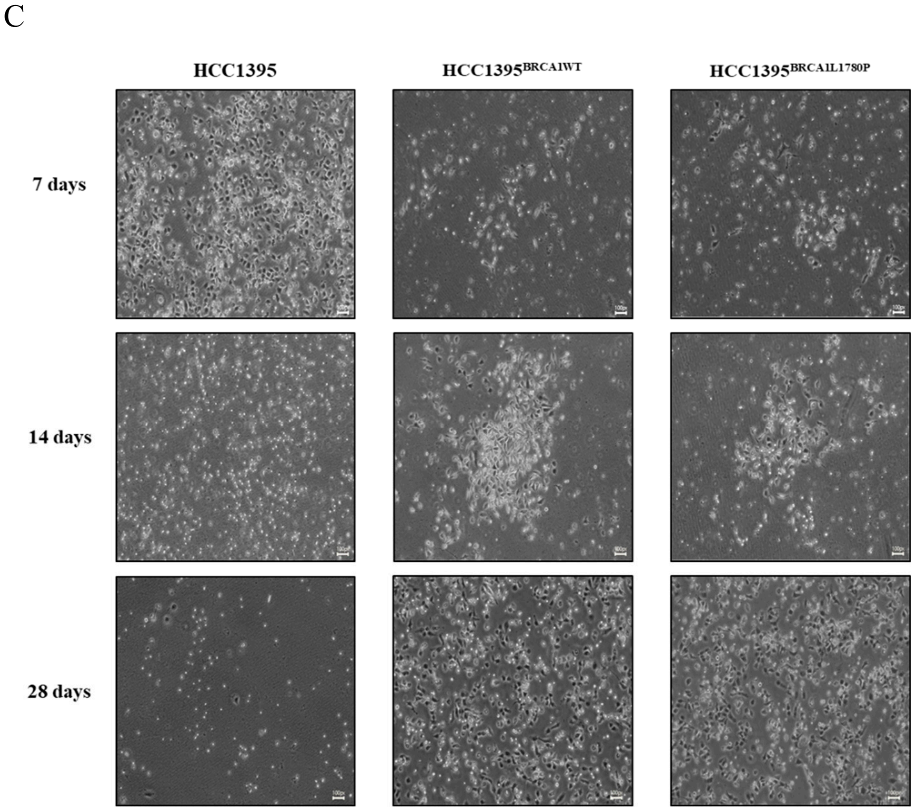

D

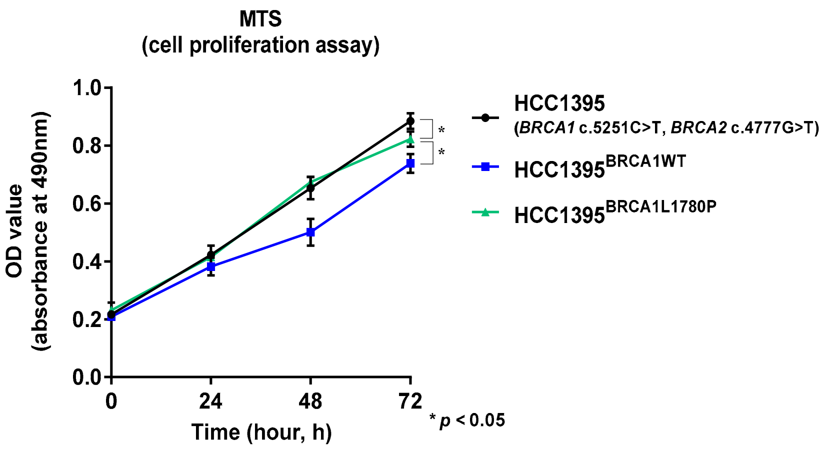

E

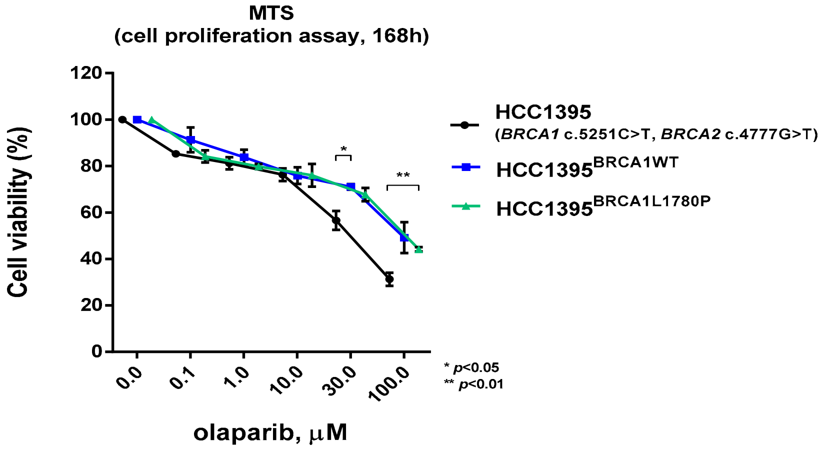

F

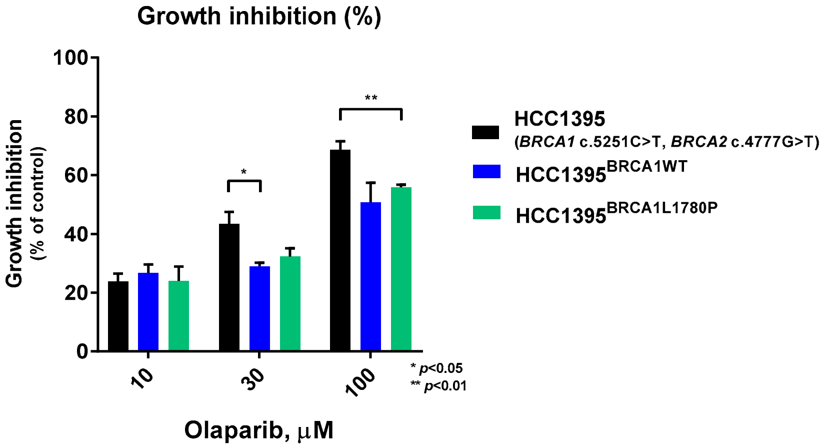

Figure S1. Stable expression of exogenous *BRCA1* wild type and L1780P mutation in HCC1395 cells.

(A) HCC1395 cells, which had *BRCA1* and *BRCA2* nonsense mutation also used to reveal the molecular

characteristics of BRCA1 L1780P mutation. (B) Anti-FLAG was detected by western blot. (C) Several time points of stably-transfected cell lines which were selected by geneticin. (D) HCC1395 parental and stably-transfected cells were used to cell proliferation assay. (E-F) Cell viability and growth inhibition following olaparib treatment in parental HCC1395 cells and stably-transfected cell lines. Data are reported as the mean ± standard deviation (SD). Statistical analysis was performed using chi-square test. (* p < 0.05, ** p < 0.01).

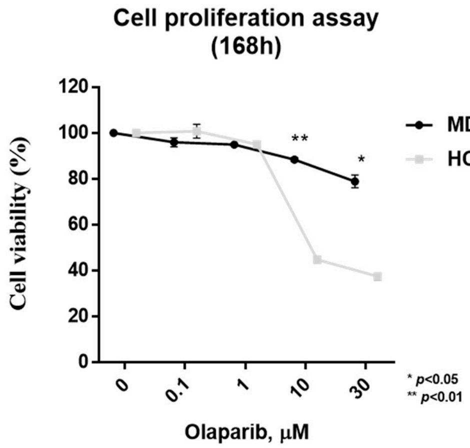

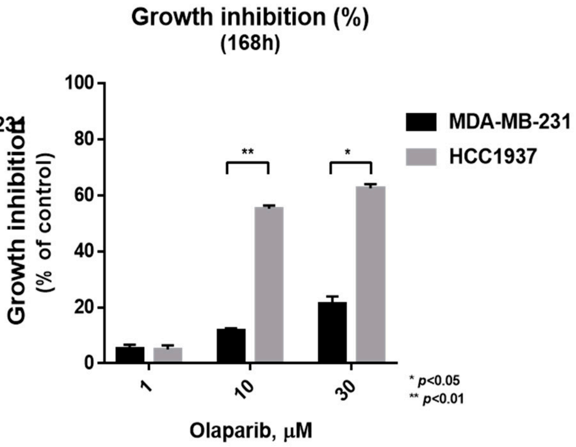

A **B**

C

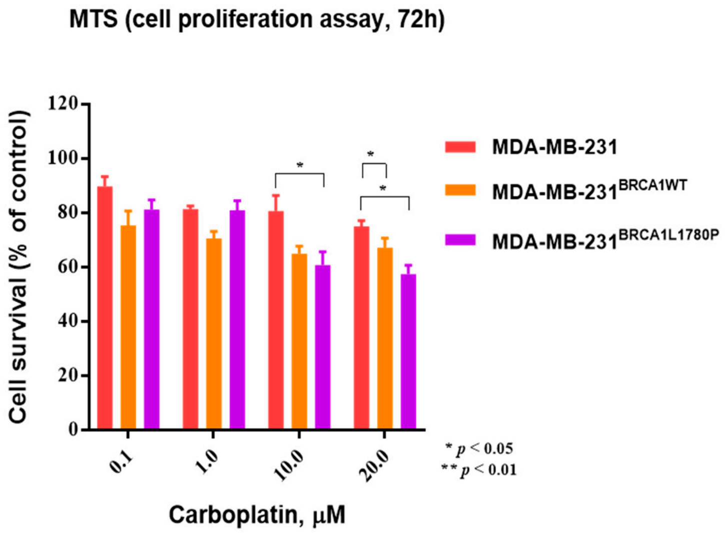

D

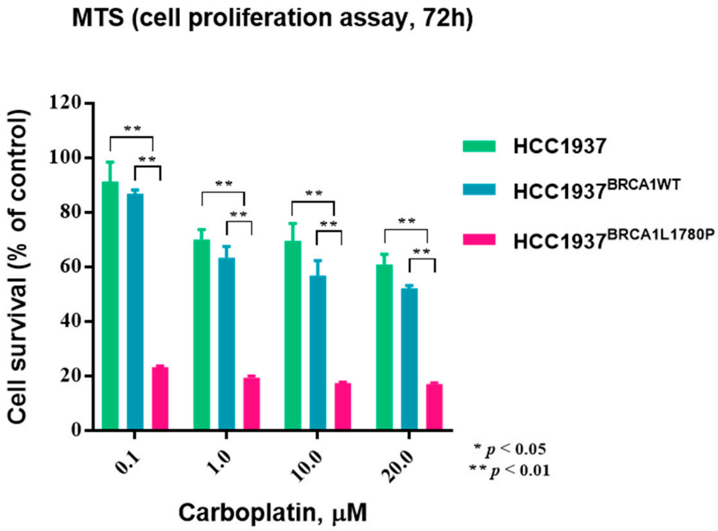

Figure S2. Olaparib and carboplatin sensitivity in parental MDA-MB-231 and HCC1937 cells. (A-B) Cell viability and growth inhibition of parental MDA-MB-231 and HCC1937 cells upon olaparib treatment. (C-D) Cell survival of parental MDA-MB-231 and HCC1937 cells and their stably-transfected cell lines upon carboplatin. Data are reported as the mean ± standard deviation (SD). Statistical analysis was performed using chi-square test. (* p < 0.05, ** p < 0.01).

A

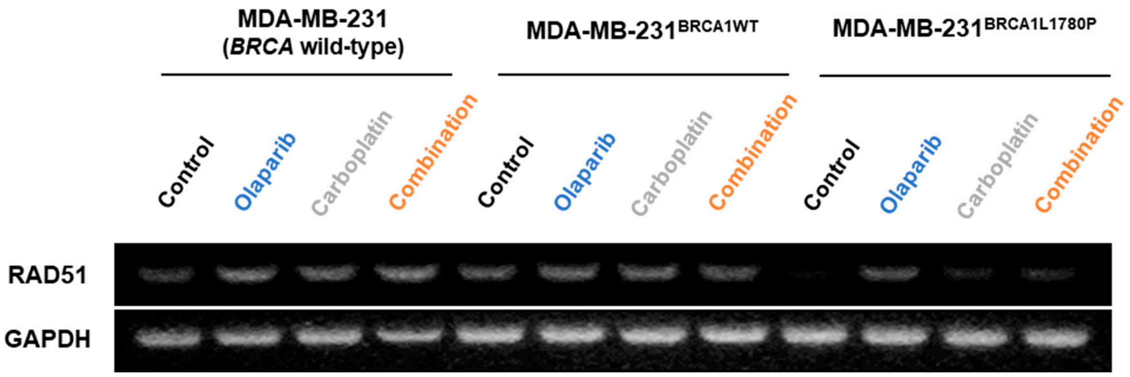

B

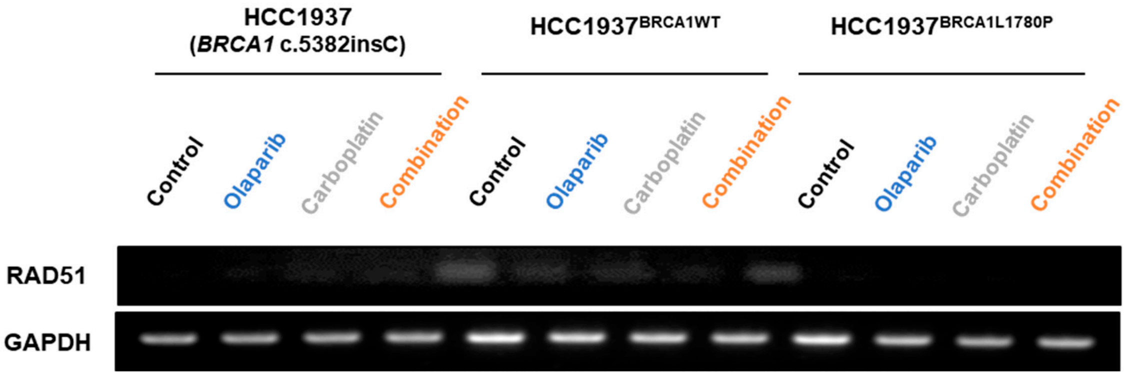

Figure S3. mRNA expression level of RAD51 was downregulated in *BRCA1* L1780P mutant cells upon combination treatment of olaparib and carboplatin. (A) MDA-MB-231 and exogenous *BRCA1* wild type cells showed normal expression of RAD51 upon olaparib and carboplatin treatment. However, *BRCA1* L1780P mutant cells showed significantly downregulated mRNA expression of RAD51. (B) HCC1937 cells, which had *BRCA1* nonsense mutation showed downregulated mRNA expression of RAD51 upon drug treatment. *BRCA1* L1780P mutant cells also showed downregulated mRNA expression of RAD51 upon drug treatment.

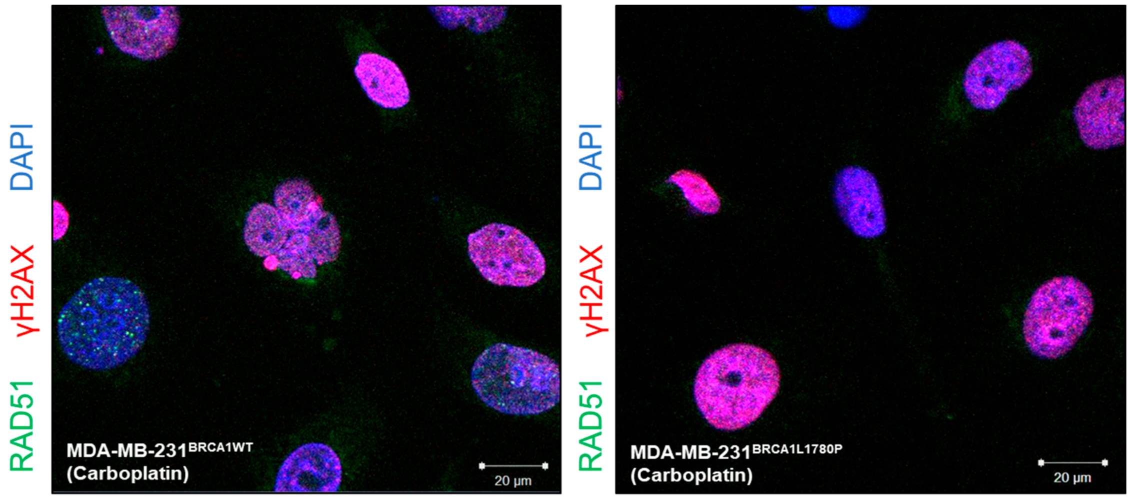

A

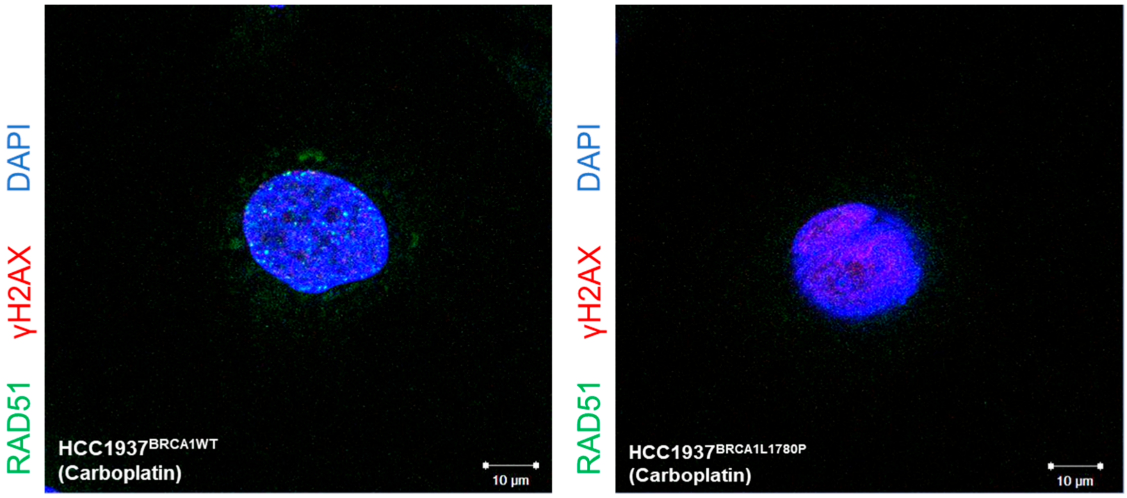

B

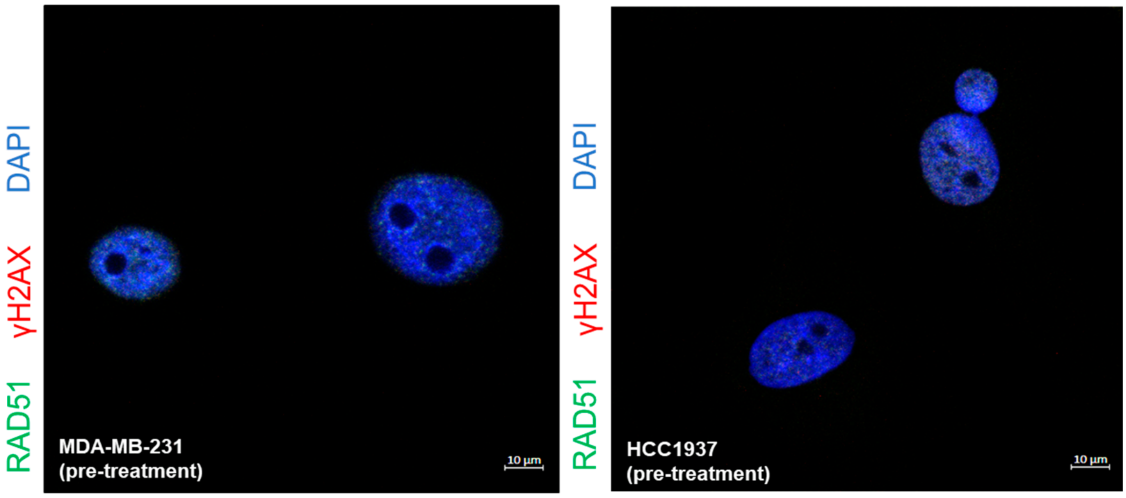

C

Figure S4. RAD51 recruitment in cell nucleus of *BRCA1* stable expressing cells. (A-B) RAD51 recruitment of cell nucleus in *BRCA1* wild type and L1780P mutant cells. Recruitment of RAD51 significantly decreased in BRCA1 L1780P mutant cells upon carboplatin treatment. (C) pre-treatment status of parental cells.

A

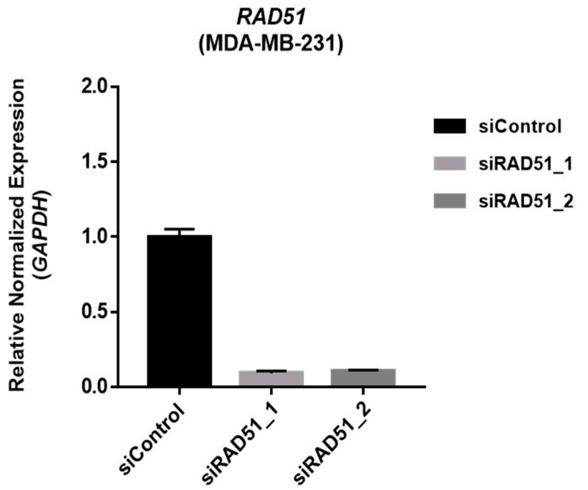

Figure S5. RAD51 knock down in MDA-MB-231 cells. (A) To evaluate the efficacy of RAD51 knock down, qRT-PCR was performed in MDA-MB-231 cells.

A

B **C**

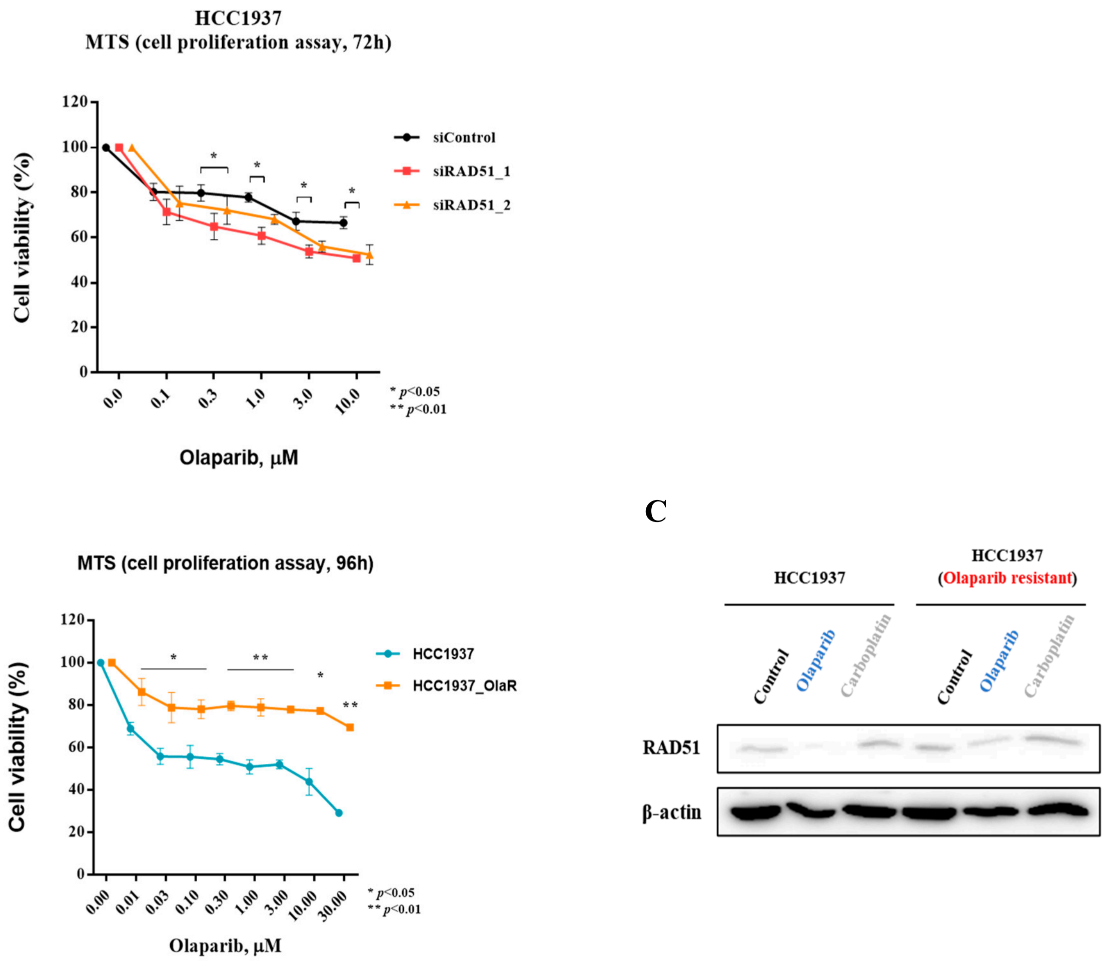

Figure S6. Downregulation of RAD51 and olaparib resistance in parental HCC1937 cells. (A) Downregulation of RAD51 expression affected sensitivity to olaparib in HCC1937 cells. (B) Olaparib-resistant cells derived from HCC1937 cells. (C) Upregulation of RAD51 expression affects olaparib resistance in HCC1937 cells. Data are reported as the mean ± standard deviation (SD). Statistical analysis was performed using the chi-square test. (* $p < 0.05$, ** $p < 0.01$).

Table S1. Primary and secondary antibodies.

**Primary antibody**	**Manufacturer**	**Catalog Number**	**Dilution**	**Application**
β-actin	Abclon	AbC-2002	1:5000	WB
p-ATM	Abcam	ab81292	1:50000	WB
ATM	Bethyl Laboratories	A300-299A	1:5000	WB
p-BRCA1	Bethyl Laboratories	A300-001A	1:500	WB
BRCA1	Bethyl Laboratories	A300-000A	1:1000	WB
RAD51	Cell Signaling Technology	#8875S	1:1000	WB
Monoclonal anti-Flag M2	Sigma Aldrich	F3165	1:2000	WB
γ-H2AX	Abcam	ab195189	1:200	IF
RAD51	Abcam	ab196449	1:100	IF
Geminin	Santa Cruz	sc-74456	1:50	IF
mounting medium with DAPI	Vector laboratories	H1200		IF

**Secondary antibody**	**Manufacturer**	**Catalog Number**	**Dilution**	**Application**
HRP conjugated anti-mouse IgG	Enzo Life Sciences	ADI-SAB-100	1:2500	WB
HRP conjugated anti-rabbit IgG	Enzo Life Sciences	ADI-SAB-300	1:5000	WB
HRP conjugated anti-goat IgG	Bethyl Laboratories	A50-101P	1:5000	WB
Mouse IgG-heavy and light chain	Bethyl Laboratories	A90-516C3	1:50	IF
Rabbit IgG-heavy and light chain	Bethyl Laboratories	A120-101F	1:50	IF
